# 556 Minimally Invasive Contracture Release Improves Function in Patients with Burn Contractures

**DOI:** 10.1093/jbcr/irad045.152

**Published:** 2023-05-15

**Authors:** Sigrid Blome-Eberwein, Adam Schwartz

**Affiliations:** Lehigh Valley Health Network, Allentown, Pennsylvania; Lehigh Valley Health Network, Allentown, Pennsylvania

## Abstract

**Introduction:**

Scar contracture bands after burns are a persistent problem that cause discomfort and physical and aesthetic limitation. Standard treatment for contracture bands in burn scars involves physical therapy, stretching, splinting and laser treatment, as well as surgical releases with local/distant flaps and grafting. A minimally invasive transcutaneous approach for the release of scar bands was initiated in our institution 6 years ago. The method was derived from techniques pioneered by Jose Daher and Raul Gonzalez to interrupt platysma bands in neck rejuvenation.

With IRB approval we evaluated this new approach regarding long term outcomes.

**Methods:**

A retrospective review of 45 patient charts was conducted for those with burn scars who received subcutaneous contracture release from May 1, 2016, through March 31, 2022. The procedure involves introducing a braided suture to loop the contracture band and moving it in a sawing motion until it cuts through the band in multiple sites along the contracture. Demographic data, total burn surface area (TBSA), location, amount of contracture release increments, scar size, and duration of treatment was recorded. Concurrent scar treatments and whether performed under anesthesia was also noted. In follow up visits 3-12 months post release patients were surveyed on satisfaction with the treatment and whether they noticed increased mobility after the procedure. The Vancouver Scar Scale was administered pre- and post-treatment. Patient range of motion was measured pre-and post treatment to assess for change in mobility at treated sites.

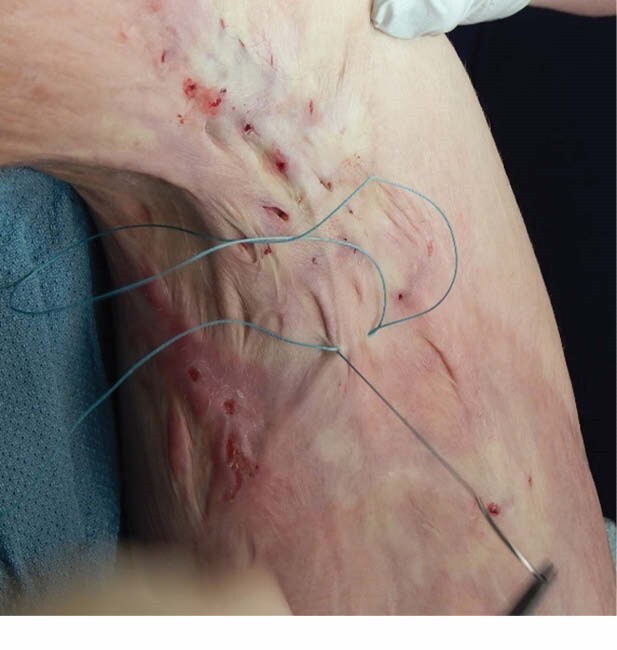

**Results:**

Male to female ratio was 51%:49%, average age was 38.8(6 to 68), 58% were white . TBSA ranged from 0.5% to 85%, with an average of 36.1 ± 22.3. The release was applied to 11 different sites spanning the entire body, 39% on the neck . The procedure involved an average of 19.0 ± 8.8 incremental releases, and the average scar size for treatment was 174.4 ± 140.0 cm^2^. 93% of the procedures involved laser treatment, 76% were done under anesthesia because of the laser, and 84% involved a site that was skin grafted. Only one of the 45 patients reported bleeding post-discharge, and none had an infection at a puncture site. Out of 20 responses, 13 patients reported being “satisfied” and 3 “very satisfied”; none reported being “dissatisfied”. 19 noted an increase in mobility at the contracture site and one did not. On average, patient range of motion increased by 13.6 degrees.

**Conclusions:**

The minimally invasive contracture release described is a versatile, safe, and well-tolerated procedure that can help patients regain form and function after a burn injury. It was successfully applied to a large range of age groups, burn sizes, and scar contracture sites. Encouraging data regarding patient recovery and gain in mobility supports the use of this contracture release technique. Further studies should include prospective multicenter trials.

**Applicability of Research to Practice:**

Immediate

